# Supplementary figures and images for: DynPeak: An Algorithm for Pulse Detection and Frequency Analysis in Hormonal Time Series
Source: PLoS One. 2012 Jul 3;7(7):e39001. doi: 10.1371/journal.pone.0039001 (PMC3389032; doi:10.1371/journal.pone.0039001)

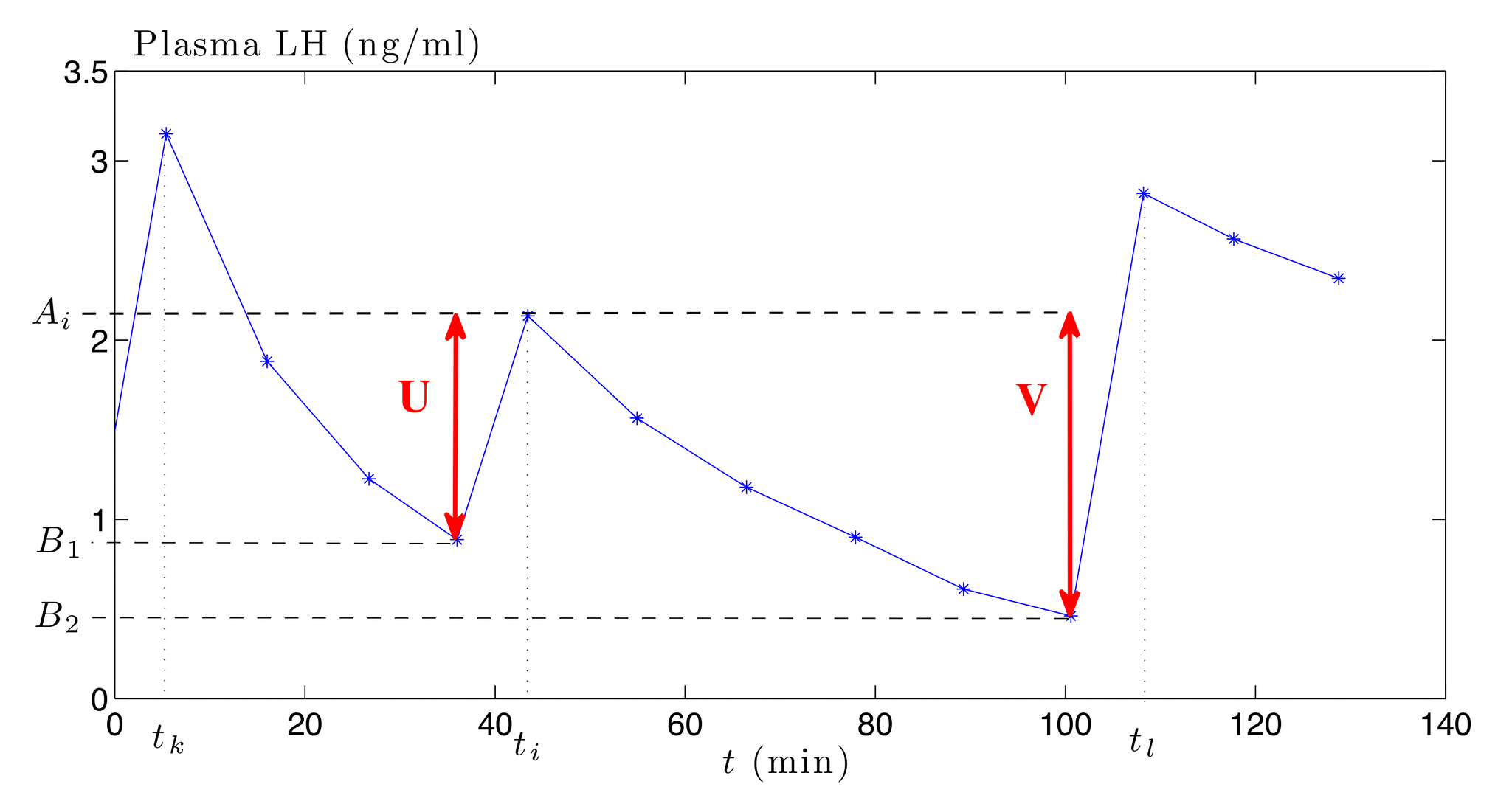

Supplement: Figure S1 — Definition of the magnitude of a peak. Let P be a given vector of potential pulses. Considering the peak occurring at , we assume that P contains the pulses just before and after occurring at and . We define U (resp. V) as the difference between the peak amplitude and the minimum value (resp. ) of the time series between and (resp. and ) : (resp. ). The magnitude of the peak occurring at is the geometric mean between U (1.4 ng/ml) and V (1.8 ng/ml). Here, the peak magnitude is equal to 1.587 ng/ml. (TIF) [file pone.0039001.s001.tif]

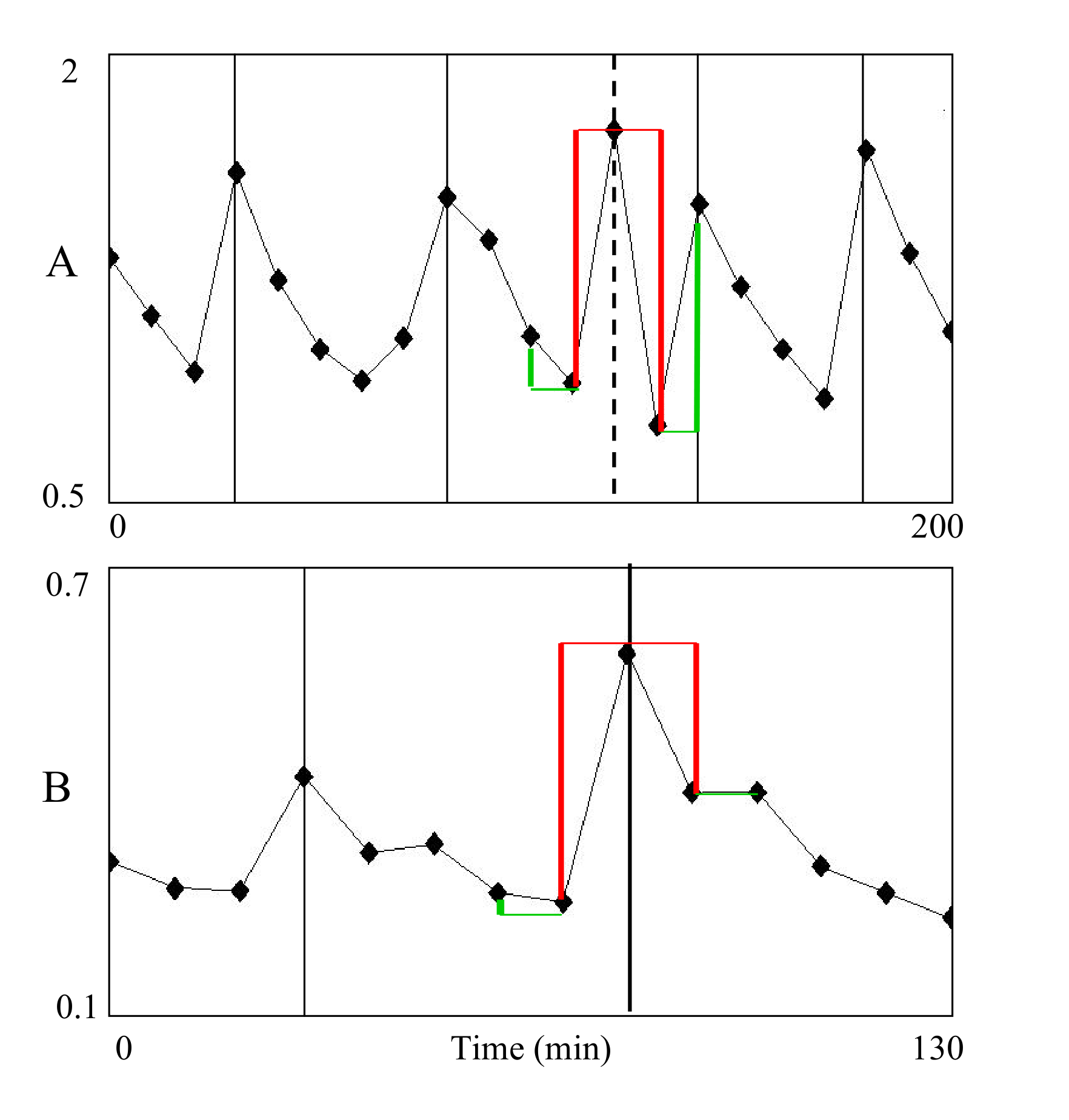

Supplement: Figure S2 — Identification of a 3-point peak pattern. Parameter corresponds to the ratio between the arithmetic mean of the amplitude of the neighboring points of rank 2 (green lines) and the geometric mean of the amplitude of the immediate neighbors (red lines). Panel A: the selected pulse (dashed vertical line) is identified as a genuine 3-point peak. Panel B: the selected pulse (solid vertical line) is not identified as a 3-point peak, since it belongs to a genuine, asymmetric LH pulse with an exponential decrease, albeit locally noised. (TIF) [file pone.0039001.s002.tif]

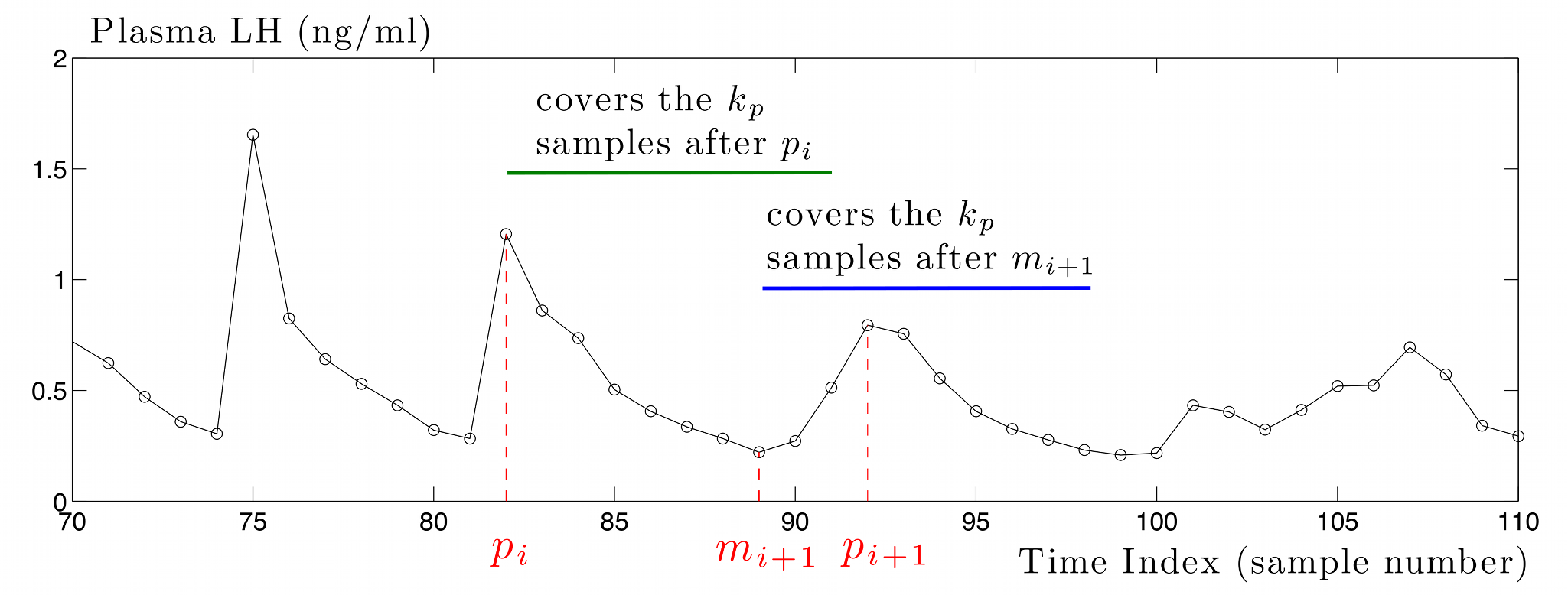

Supplement: Figure S3 — One iteration of the forward research of pulses. For a given value of i in the iterative process (initialized with i = 1), the algorithm searches for the index of the minimal sample from the sample in the window defined by the nominal period Tp, i.e. among the kp samples (under the green segment) directly following the sample. Then, the algorithm searches for the index of the maximal sample among the kp samples (under the blue segment) directly following the sample. Index is stored in vector P and the process is iterated with i incremented by 1 until the end of the time series. (TIF) [file pone.0039001.s003.tif]

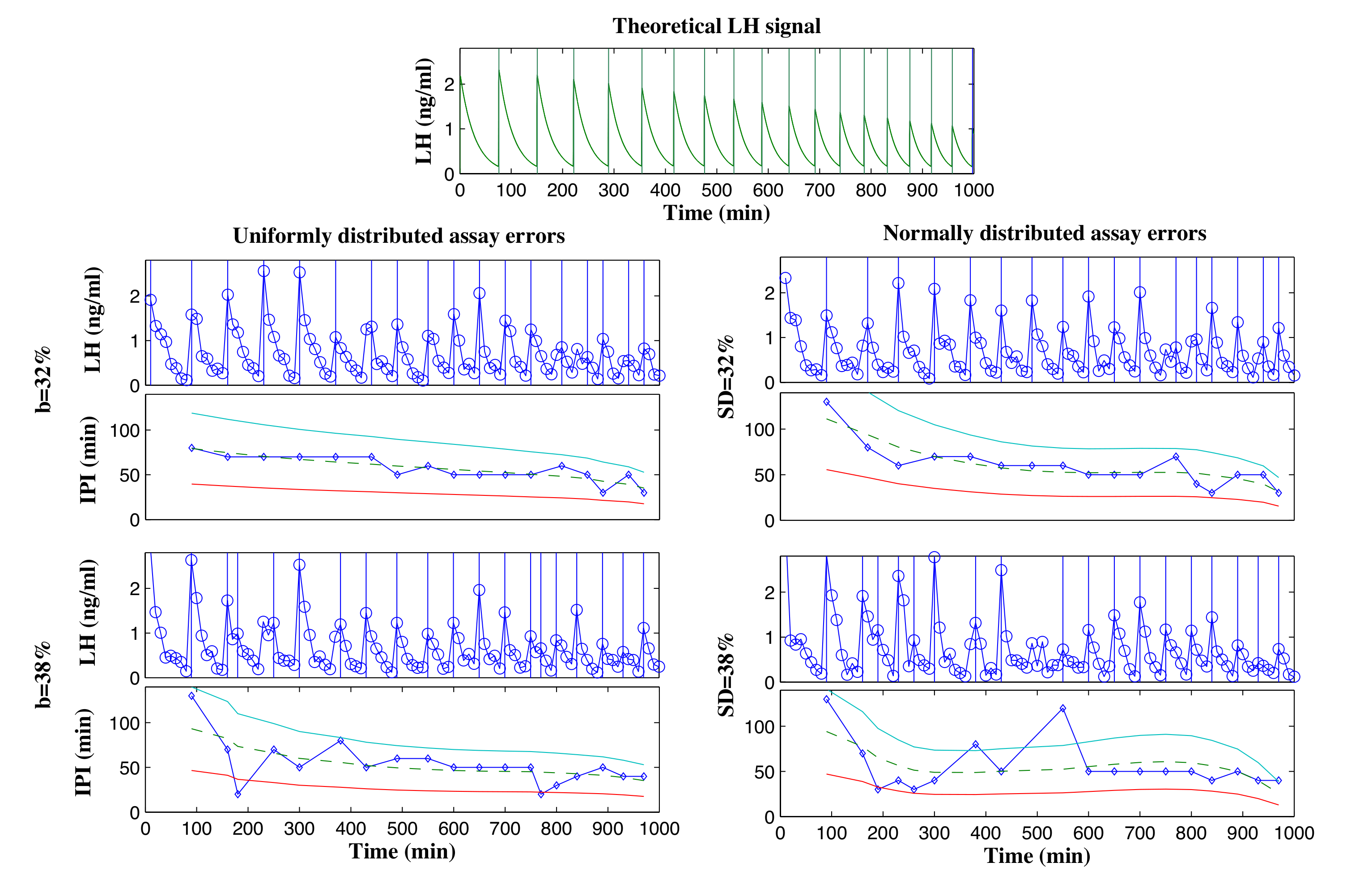

Supplement: Figure S4 — Outputs of the algorithm applied to synthetic LH time series obtained with uniformly distributed or normally distributed assay errors. Top panel: theoretical continuously measured LH blood level (green curve) obtained with a spike amplitude function decreasing linearly from 15 to 6.5 ng/ml and an interspike interval function decreasing linearly from 80 to 50 min. The pulse times are highlighted by vertical green bars. The other panels represent the outputs of the algorithm for 4 time series obtained with either uniformly (b = 32 or 36%) or normally (SD = 32 or 36%) distributed assay errors and a sampling period Ts = 10 min. In each case, the upper panel represents the time series (blue circles) with the detected pulse occurrences (vertical blue bars) and the lower panel displays the detected IPI series (blue diamonds) together with the IPI tunnel (delimited by the cyan and red lines). In both cases where b and SD equal to 32%, the algorithm has detected the pulses of the time series accurately. In the case of a uniform distribution with amplitude b = 38%, two over-detections occurred around 180 min and 770 min. Both led to IPI outliers. In the case a normal distribution with SD = 38%, two over-detections occurred around 190 min and 270 min and a lack of detection occurred around 500 min. Both the first over-detection and the lack of detection implied IPI outliers, yet the IPI sequence remains in the tunnel, even if it is close to the lower edge, near 270 min. (TIF) [file pone.0039001.s004.tif]
