# Supplementary material for: DynPeak: An Algorithm for Pulse Detection and Frequency Analysis in Hormonal Time Series
Source: PLoS One. 2012 Jul 3;7(7):e39001. doi: 10.1371/journal.pone.0039001 (PMC3389032; doi:10.1371/journal.pone.0039001)
Supplement: Table S1 — Accuracy of the algorithm outputs for synthetic LH time series according to the features of the assay error distribution. The same set of 6 different values for the amplitude b of the uniform distribution (UD) of the assay error and for the standard deviation SD of the normal distribution (ND) of the assay error has been used to extract time series with a 10 minutes sampling period. In each case (column), 10 time series have been generated from the same theoretical LH signal with decreasing pulse amplitude and interpulse interval (see top panel of Figure S4). The table displays the corresponding numbers of time series for which the detection algorithm (i) detected all the pulses accurately, (ii) missed a pulse or produced an over-detection leading to IPI outlier or (iii) missed a pulse or produced an over-detection without an associated IPI outlier. (PDF) [file pone.0039001.s005.pdf]

	UD assay error						ND assay error					
b value (for UD) or SD value (for ND)	20 %	25%	30%	32%	38%	40%	20%	25%	30%	32%	38%	40%
Well-detected time series	10	9	8	6	3	2	10	10	8	5	2	1
Over- or under-detection with IPI outlier	0	1	2	4	6	8	0	0	2	5	7	8
Over- or under-detection without IPI outlier	0	0	0	0	1	0	0	0	0	0	1	1
