# Supplementary material for: DynPeak: An Algorithm for Pulse Detection and Frequency Analysis in Hormonal Time Series
Source: PLoS One. 2012 Jul 3;7(7):e39001. doi: 10.1371/journal.pone.0039001 (PMC3389032; doi:10.1371/journal.pone.0039001)
Supplement: Appendix S1. — (PDF) [file pone.0039001.s006.pdf]

Appendix

Algorithm description

Overview of the algorithm. Besides the time series of N samples and the sampling period Ts, the user is asked to enter values of four parameters that rule the acuteness of the detection process and two parameters that define the width of the IPI tunnel. We use the parameter names in the following algorithm description. We give a tutorial in the result section for helping the users to choose the appropriate values.

1. The nominal period Tp represents the smallest duration in which, from one pulse occurrence, one expects the following one. In what follows, we note:

$$kp = \left\lfloor \frac{Tp}{Ts} \right\rfloor .$$

   Note that the time interval $[t_{k+1}, t_k + Tp]$ contains the kp time indexes $\{k+1, ..., k+kp\}$.

2. The relative magnitude threshold: λ_r,
3. the absolute magnitude threshold ratio: λ_a,
4. the relative magnitude threshold ratio for 3-point peaks: λ_{3p},
5. the ratios defining the edges of the IPI tunnel: α, β.

The algorithm body is segmented into 6 steps. The third step is itself segmented into 3 substeps. Each step is described in detail below with an accompanying box enunciating the corresponding pseudo-code.

The output of the algorithm consists in:

1. the set P of detected pulse occurrences,
2. the corresponding sequence Θ of IPIs,
3. the lower and upper edges of the IPI tunnel, ψ_{inf} and ψ_{sup} respectively,

and associated graphical outputs (of which we make an intensive use in the result section):

1. the sampled time series $(A_i)_{i=1}^n$ versus the sampling times with identified pulse occurrences,
2. the graph of the IPI versus the corresponding pulse occurrences, more precisely $(p_{i+1}, \theta_i)_{i=1}^{s(P)-1}$, together with the IPI tunnel.

Input data: $(A_i)_{i=1}^n, Ts, Tp, \lambda_r, \lambda_a, \lambda_{3p}, \alpha, \beta$.

Algorithm step 1

⋮

Algorithm step 6

Output data: $P, \Theta, \psi_{\text{inf}}, \psi_{\text{sup}}$.

Related graphical outputs:

- the time series $(t_i, A_i)_{i=1}^n$ with vertical bars indicating the detected pulse occurrences,
- the graph of the IPI versus the corresponding pulse occurrences and the IPI tunnel: $(p_{i+1}, \theta_i)_{i=1}^{s(P)-1}, \psi_{\text{inf}}$ and ψ_{sup}.

Notations In the following, we search iteratively for a time index corresponding to the minimal (resp. maximal) value of a subset of time series A_k. When there are several time indexes verifying this condition, we choose the smallest one. Hence, for I a subset of {1,2, ..., N}, we define the following notations:

$$\arg\min_{k\in I} A_k = \min\{j \in I | A_j = \min_{k\in I} A_k\}$$
$$\arg\max_{k\in I} A_k = \min\{j \in I | A_j = \max_{k\in I} A_k\}$$

Step 1: Search for the first pulse. Under the assumption that the maximum value of A_k retrieved from a sufficiently large time window coincides with a pulse occurrence, the first pulse is detected by searching for the maximum value of A_k for k in {1,2,...,2kp} where $kp = \lfloor Tp/Ts \rfloor$. The choice of the time window size (twice nominal IPI) containing $2kp$ samples is a trade-off between the risk of missing the first pulses (encountered when the window is too large) and the risk of false detection (encountered when the window is too small).

Step 1: Find the first pulse occurrence index p_1 by searching for the maximum value of the sampled time series A_k within the first $2kp$ data samples:

$$p_1 := \arg\max_{k\in\{1,2,\dots,(2kp)\}} A_k$$

Step 2: Search for the pulses following the first one. After the detection of the first pulse located at $k = p_1$, the second one could be detected by searching for the maximum value of A_k for $k \in \{(p_1+1),(p_1+2),\dots,(p_1+kp+s)\}$, where some margin value s should be chosen for the trade-off between over-detection and under-detection. To reduce the risks of misdetection, instead of searching for the maximum value of A_k at this stage, the minimum value of A_k for $k \in \{(p_1+1),\dots,(p_1+kp)\}$ is first looked for. Let $k = m_2$ be the location of this minimum, then the second pulse is searched for within the window $\{(m_2+1),\dots,(m_2+kp)\}$. These searches are made within windows of size kp (see Figure S3). As the minimum and the maximum being searched for are expected to be inside the windows of size kp, there is no need to choose a margin value. The following pulses are then searched for similarly.

Step 2: Find the following pulse occurrences p_i by alternatively searching for the minimum and maximum values of the sampled time series in a moving window covering kp data samples:

$$i := 1$$

while $p_i + kp \leq N$ **do**

$$m_{i+1} := \arg\min_{k\in\{p_i+1,p_i+2,\dots,p_i+kp\}} A_k$$

if $m_{i+1} + kp \leq N$ **then**

$$p_{i+1} := \arg\max_{k\in\{m_{i+1}+1,m_{i+1}+2,\dots,m_{i+1}+kp\}} A_k$$

end if

$$i := i + 1$$

end while

$$s_p := i - 1$$
$$P := \{p_1,\dots,p_{s_p}\}$$

Step 3: Remove too small peaks by various thresholding methods.

Step 3.1: Pulse height median-based thresholding. Several methods are used to remove small pulses possibly resulting from false detections. The first method is based on a threshold applied to the heights of the detected pulses. We recall that the height of the i-th pulse is defined as the difference between its amplitude A_{p_i} and the lowest value of A_k within the sampled time series, denoted by $\underline{A}$. If the height of a pulse is lower than the median of the heights of all the detected pulses multiplied by a ratio λ_r, then it is removed from the set of detected pulses.

Step 3.1. Pulse height median-based thresholding:

$$\underline{A} := \min_{k \in 1,2,\ldots,N} A_k$$
$$P := \{p_i \in P : A_{p_i} - \underline{A} > \lambda_r(\text{median}(A_{p_1}, \ldots, A_{p_{s_p}}) - \underline{A})\}$$
$$s_p := s(P)$$

Step 3.2: Local relative magnitude-based thresholding. The second method for removing false pulses is to compare the magnitude of each pulse with those of its neighbors. Let B_1 and B_2 be respectively the minimum values of A_k for $p_{i-1} < k < p_i$ and $p_i < k < p_{i+1}$. The local magnitude of the i-th pulse is defined as the geometric mean of $(A_{p_i} - B_1)$ and $(A_{p_i} - B_2)$. Let B_0 be the lowest value out of B_1 and B_2, The local magnitude of the i-th pulse is compared with the geometric mean of $(A_{p_{i-1}} - B_0)$ and $(A_{p_{i+1}} - B_0)$, relatively to a threshold λ_r. The comparison of geometric means is equivalent to the comparison of arithmetic means in the logarithmic scale, so that it tends to favor smaller quantities.

Step 3.2. Local relative magnitude-based thresholding:
for $i = 2, \ldots, (s_p - 1)$ **do**

$$B_1 := \min_{k \in \{(p_{i-1}+1),(p_{i-1}+2),\ldots,(p_i-1)\}} A_k$$
$$B_2 := \min_{k \in \{(p_i+1),(p_i+2),\ldots,(p_{i+1}-1)\}} A_k$$
$$B_0 := \min(B_1, B_2)$$

if $(A_{p_i} - B_1)(A_{p_i} - B_2) < \lambda_r^2(A_{p_{i-1}} - B_0)(A_{p_{i+1}} - B_0)$ **then**
Erase p_i from P
end if
end for

$$s_p := s(P)$$

Step 3.3: Local magnitude absolute thresholding. The third method for removing false pulses is based on some prior knowledge about the magnitudes of the pulses. Let B_1 and B_2 be respectively the minimum values of A_k for $p_{i-1} < k < p_i$ and $p_i < k < p_{i+1}$. If the local magnitude of the i-th pulse (the geometric mean of $A_{p_i} - B_1$ and $A_{p_i} - B_2$) is smaller than a chosen threshold λ_a, then it is removed from the set of detected pulses.

Step 3.3. Local magnitude absolute thresholding:
for $i = 2, \ldots, (s_p - 1)$ **do**

$$B_1 := \min_{k \in \{(p_{i-1}+1),(p_{i-1}+2),\ldots,(p_i-1)\}} A_k$$

$$B_2 := \min_{k \in \{(p_i+1),(p_i+2),\ldots,(p_{i+1}-1)\}} A_k$$

  if $(A_{p_i} - B_1)(A_{p_i} - B_2) < \lambda_a^2$ **then**
    Erase p_i from P
  end if
end for

$$s_p := s(P)$$

Step 4: Retrieve missed pulses. To retrieve possible missed pulses, the data points lying between each pair of detected pulses are examined. For each sample index j lying between the pulse occurrences p_i and p_{i+1}, the relative height of the sample A_j is defined as the geometric mean of $A_j - B_1$ and $A_j - B_2$, where B_1 and B_2 are respectively the lowest values of A_k for $p_i < k \leq j$ and for $j \leq k < p_{i+1}$. The sample exhibiting the maximum relative height between p_i and p_{i+1} is compared with the geometric mean of $A(p_i) - B_0$ and $A(p_{i+1}) - B_0$, where B_0 is the lowest sample value between p_i and p_{i+1}. If the comparison with respect to the threshold λ_r is conclusive, a new pulse is added to the set of detected pulses. This process is repeated three times to deal with the case where several pulses might have been missed between two consecutive previously detected pulses..

Step 4. Retrieve missed pulses.
for $k = 1, 2, 3$ **do**
  $J = \emptyset$
  for $i = 1, \ldots, (s_p - 1)$ **do**
    if $p_i + 3 < p_{i+1}$ **then**
      for $j = (p_i + 2), \ldots, (p_{i+1} - 2)$ **do**

$$B_1 := \min_{k \in \{(p_i+1), \ldots, j\}} A_k$$
$$B_2 := \min_{k \in \{j, \ldots, (p_{i+1}-1)\}} A_k$$
$$C_j := (A_j - B_1)(A_j - B_2)$$

      end for

$$j_{\max} := \arg \max_{j \in \{(p_i+2), \ldots, (p_{i+1}-2)\}} C_j$$
$$B_0 := \min(B_1, B_2)$$

      if $C_{j_{\max}} > \lambda_r^2 (A(p_i) - B_0)(A(p_{i+1}) - B_0)$ **then**
        Insert $j_{\max}$ into J
      end if
    end if
  end for
  Insert J into P
  $s_p = s(P)$
end for

Step 5: Removal of 3-point peaks. When the rhythm of the hormonal pulses accelerates, the IPI may approach the sampling period Ts, to the point that it becomes impossible to reliably detect pulses. In such situations, the sampled time series may exhibit local maxima supported by 3 consecutive data samples. To avoid false detections, pulses detected upon 3 points are identified and possibly removed. For a pulse detected at $k = p_i$, if $p_i - 1$ and $p_i + 1$ are local minima "sharp" enough, in the sense that $A(p_i - 2) - A(p_i - 1)$ and $A(p_i + 2) - A(p_i + 1)$ are large enough compared to $A(p_i) - A(p_i - 1)$ and $A(p_i) - A(p_i + 1)$, then A_{p_i} is considered as a peak detected upon 3 points (referred to as "3-point peak") and is removed from the set of detected pulses.

Step 5. Remove pulses detected upon 3 data points:

$$s_p := s(P)$$

for $i = 1, \ldots, s_p$ **do**

$$\textbf{if} \left\{ \begin{array}{llll} & p_i \geq 3, & \text{and} & p_i \leq N - 2, \\ \text{and} & A(p_i - 2) > A(p_i - 1), & \text{and} & A(p_i) > A(p_i - 1), \\ \text{and} & A(p_i) > A(p_i + 1), & \text{and} & A(p_i + 2) > A(p_i + 1) \end{array} \right\} \textbf{then}$$

$$R := \frac{\{[A(p_i - 2) - A(p_i - 1)] + [A(p_i + 2) - A(p_i + 1)]\}/2}{\sqrt{[A(p_i) - A(p_i - 1)][A(p_i) - A(p_i + 1)]}}$$

if $R \geq \lambda_{3p}$ **then**
Erase p_i from P
end if
end if
end for

Step 6: IPI sequence and tunnel construction. Under some regularity assumptions of the IPIs, the detected pulses leading to IPI outliers should be corrected. For this purpose, a cubic polynomial $\theta_0 + \theta_1 x + \theta_2 x^2 + \theta_3 x^3$ is first fitted to the IPIs where x is equal to the shifted pulse index i. Let $(\hat{\theta}_0, ..., \hat{\theta}_3)$ be the value of $(\theta_0, ..., \theta_3)$ fitted to the segmented IPIs and $\phi(x) = \hat{\theta}_0 + \hat{\theta}_1 x + \hat{\theta}_2 x^2 + \hat{\theta}_3 x^3$. The lower and upper edges of the IPI tunnel are defined respectively by $\phi\left(x + \frac{s_p - 1}{2}\right)(1 - \alpha)$ and $\phi\left(x + \frac{s_p - 1}{2}\right)(1 - \beta)$. The centering of the i-indexes (leading to x_i) is for the purpose of a better numerical accuracy.

Step 6. IPI sequence and tunnel construction:

$$s_p := s(P)$$

for $i = 1, \ldots, s_p - 1$ **do**

$$q_i := p_{i+1} - p_i$$

$$x_i := i - \frac{s_p - 1}{2}$$

end for

$$(\hat{\theta}_0, \hat{\theta}_1, \hat{\theta}_2, \hat{\theta}_3) = \arg \min_{(\theta_0, \ldots, \theta_3) \in \mathbb{R}^4} \sum_{i=1}^{s_p - 1} \left[(\theta_0 + \theta_1 x_i + \theta_2 x_i^2 + \theta_3 x_i^3) - q_i\right]^2$$

Algorithm robustness to assay error

In our model of synthetic LH time series presented in the Methods section, we have introduced a multiplicative noise characterized by the random numbers $\rho_{assay}(i) \in [-b, b]$ (see Equation 5 of the manuscript) obtained from a quasi-uniform distribution. Of course, it is also possible to choose another statistical distribution, such as the normal distribution with a prescribed standard deviation SD, to reproduce the assay variance. We have run several tests to investigate the robustness of our algorithm on several trials differing by the type of distribution (either uniform or normal), the maximum amplitude (in the uniform case) or SD value (in the normal case) and the absence or introduction of dose-dependence.

We have assessed the ability of our algorithm to detect LH pulses in synthetic time series built with a high, dose-independent variability. We have investigated both uniformly distributed (UD) and normally distributed (ND) assay errors using the same set of values for the amplitude b of the uniform distribution and the standard deviation SD of the normal distribution: 25%, 30%, 32%, 38% and 40%. In each case, we have generated 10 synthetic LH time series by sampling, every 10 min, the theoretical signal with the same pattern of decreasing pulse amplitude and decreasing interpulse interval as in case F of Figure 6. We have applied our algorithm with the default parameter values and we have analyzed its output for each time series. Table S1 gives the corresponding number of time series, over the 10, for which the outputs of the detection algorithm fall in one of the 3 following cases :

1. detection of every pulse duly associated with a spike, but no over-detection of artifact LH pulse (well-detected time series),
2. detection of an extra, artifact pulse (over-detection) or missing of a genuine pulse (under-detection), with a repercussion on the IPI series (IPI outlier),
3. over-detection or under-detection, without identifiable repercussion on the IPI series (no IPI outlier).

To illustrate these results, we have shown, in Figure S4, representative algorithm outputs obtained from 4 instances of simulated LH time series with either UD assay errors (b = 32 % and b = 38 %) or ND assay errors (SD = 32% and SD = 38 %) respectively.

As shown in Table S1, for b = 20 % or SD = 20 %, the algorithm detected all the 20 time series correctly. For b = 25 % or SD = 25 %, the algorithm failed rarely (1 case out of ten with UD assay errors and none with ND assay errors). For b = 30 % or SD = 30 %, the algorithm remained reliable most of the time: it failed twice in each distribution case, yet it detected the whole 16 other time series accurately. It seems that over the critical b (resp. SD) value of 30%, the algorithm becomes more sensitive to the assay variability: due to the high level of noise, the results are deprecated with b and SD set to 32%, even if they remain relevant in half cases. Instances of time series obtained with b = 32% and SD = 32% and the corresponding outputs of the algorithm (time series together with the detected pulse occurrences and IPI series) where the detection goes well are shown in Figure S4. Over- or under-detections appear in most of the outputs when the algorithm is applied to time series obtained with values of b or SD above 35%. However, it is worth noticing that, even for extreme values of b or SD (38% or 40%), over- or under-detections lead almost each time to IPI outliers (see bottom panels of Figure S4) that can be handled in the same way as we do in the Results section.
